# Comparison of in vitro activities of newer triazoles and classic antifungal agents against dermatophyte species isolated from Iranian University Hospitals: a multi-central study

**DOI:** 10.1186/s12941-023-00564-4

**Published:** 2023-02-20

**Authors:** Parisa Badiee, Tahereh Shokohi, Jamal Hashemi, Rasoul Mohammadi, Mohammad Javad Najafzadeh, Maneli Amin Shahidi, Fatemeh Ghasemi, Hadis Jafarian

**Affiliations:** 1 Clinical Microbiology Research Center, Shiraz University of Medical Sciences, Shiraz, Iran; 2 Department of Medical Mycology, School of Medicine, Invasive Fungi Research Centre (IFRC), Communicable Diseases Institute, Mazandaran University of Medical Sciences, Sari, Iran; 3 Medical Mycology Department, School of Public Health Research, Tehran University of Medical Sciences, Tehran, Iran; 4 Department of Medical Parasitology and Mycology, School of Medicine, Infectious Diseases and Tropical Medicine Research Center, Isfahan University of Medical Sciences, Isfahan, Iran; 5 Department of Medical Parasitology and Mycology, Mashhad University of Medical Sciences, Mashhad, Iran

**Keywords:** Dermatophytes, Luliconazole, *Trichophyton mentagrophytes*, *Trichophyton simii*

## Abstract

**Background:**

Dermatophytes have the ability to invade the keratin layer of humans and cause infections. The aims of this study were the accurate identification of dermatophytes by Polymerase Chain Reaction-Restriction Fragment Length Polymorphism method and sequencing and comparison between the in vitro activities of newer and established antifungal agents against them.

**Methods:**

Clinical specimens of patients from five Iranian university laboratories were entered in this study. Samples were cultured on sabouraud dextrose agar medium. For molecular identification, extracted DNAs were amplified by the universal fungal primers ITS1 and ITS4, and digested with *Mva*I enzymes. The antifungal susceptibility test for each isolate to terbinafine, griseofulvin, caspofungin, fluconazole, itraconazole, luliconazole, and isavuconazole was performed, according to the microdilution CLSI M38-A2 and CLSI M61 standard methods.

**Results:**

Two hundred and seven fungi species similar to dermatophytes were isolated of which 198 (95.6%) were dermatophytes by molecular assay. The most commonly isolated were *Trichophyton mentagrophytes* (76/198), followed by *Trichophyton interdigitale* (57/198), *Trichophyton rubrum* (34/198), *Trichophyton tonsurans* (12/198), *Microsporum canis* (10/198), *Trichophyton simii* (3/198), *Epidermophyton floccosum* (3/198), *Trichophyton violaceum* (2/198), and *Trichophyton benhamiae* (1/198). The GM MIC and MIC_90_ values for all the isolates were as follows: terbinafine (0.091 and 1 μg/ml), griseofulvin (1.01 and 4 μg/ml), caspofungin (0.06 and 4 μg/ml), fluconazole (16.52 and 32 μg/ml), itraconazole (0.861 and 8 μg/ml), isavuconazole (0.074 and 2 μg/ml), and luliconazole (0.018 and 0.25 μg/ml).

**Conclusion:**

*Trichophyton mentagrophytes*, *Trichophyton interdigitale*, and *Trichophyton rubrum* were the most common fungal species isolated from the patients. luliconazole, terbinafine, and isavuconazole in vitro were revealed to be the most effective antifungal agents against all dermatophyte isolates.

## Background

Dermatophytosis is a major public health concern, generally caused by cutaneous infections. Although these infections are chronic, they may invade deeper tissues, particularly in immunocompromised patients [1]. Dermatophytes spread through direct contact with infected humans, animals, and soil [2]. These organisms have the ability to invade the stratum corneum in the skin, hair, and nails (keratinized tissues) and are generally referred to as ‘ringworm’ or ‘tinea’ [3, 4]. The epidemiology of dermatophytosis is related to population density factors and migration, socio-economic status, climate, and environmental factors like humidity and types of activity in each region [5]. The varied prevalence rates of dermatophytosis were reported worldwide, ranging from 14 to 26.8% in North America, East Asia, and Europe and 5–31.6% in Africa (Ethiopia, Kenya, Nigeria, and Tanzania) [6–8].

The accurate identification of etiologic agents is important for appropriate treatment and control of environmental sources of infection [9]. The routine diagnosis of dermatophytosis is based on microscopic examination and culture. The former from the lesion samples is relatively insensitive and non-specific but rapid; however, the identification of some atypical isolates by culture method can be very slow and may take weeks to produce an exact result [10]. Also, morphological identification of dermatophyte species in cultures is sometimes difficult because there are overlapping character variations between species. Besides, some molecular methods can quickly identify dermatophytes at the species level directly in clinical samples or in culture [11, 12]. Analysis of the ITS regions of the ribosomal DNA gene (encoding the 5.8S rDNA) by PCR–RFLP is used as a reliable and sensitive marker for dermatophyte species identification [13, 14].

Epidemiological pattern of drug resistance in any region can helps to choose more efficacious antifungal agents for standard treatment. There is limited data available regarding in vitro antifungal susceptibility patterns of dermatophytes. Azole resistance in dermatophytes has been reported to be as high as 19% worldwide [15]. Developing azole resistance may be due to repeated exposure to azole antifungals. Resistance to griseofulvin (GRI) therapy in patients with *Trichophyton* (T) *rubrum* and *T. tonsurans* was reported in the 1960s [16]. Treatment failures in chronic infections due to *T. rubrum* were reported [17, 18]. The present study seeks the accurate identification of the dermatophyte strains isolated from Iran by PCR–RFLP method and sequencing and comparison between in vitro activities of the two newer triazoles, isavuconazole (ISA) and luliconazole (LUL), and five classic antifungal agents against them. The minimal inhibitory concentrations (MIC) of the isolates to seven antifungal agents were evaluated by CLSI M38-A2 and M61 standard methods [19, 20].

### Methods

In this cross-sectional study, dermatophyte species isolates from five medical university hospital labs in Iran (i.e. Shiraz, Isfahan, Mashhad, Sari, Tehran) were evaluated from 2019 to 2021.

### Sample collection

The inclusion criterion of species was being isolated from patients suffering from dermatophytosis. Specimens including; skin, hair, and nail scrapings, were cultured on sabouraud dextrose agar medium (Merck, Germany) and incubated at 24 °C until colony morphology was completed (about four weeks). The isolated dermatophyte species were transferred to Professor Alborzi Clinical Microbiology Research Center, Shiraz University of Medical Sciences, Shiraz, Iran. Species were re-cultured on sabouraud dextrose agar and Mycobiotic agar plate (Sigma–Aldrich, St. Louis, MO, USA) at room temperature. Macroscopic and microscopic characteristics of each colony were studied by lactophenol cotton blue smear. The cultivated fungi were identified by molecular methods (PCR–RFLP).

### Molecular assay

In a 2 ml Eppendorf tube, a small portion of the pure hyphal plug with 300 μl of lysis buffer containing; 200 mM Tris–HCl; pH 7.5 (Merck, Germany), 25 mM ethylene-diamine-tetra-acetic acid (Merck, Germany), 0.5% w/v sodium dodecyl sulfate (Sinaclon, Iran), and 250 mM NaCl (Merck, Germany) were ground with sonicator for 10 s. 300 μl of phenol–chloroform (Merck, Germany) was added and the mixture vortexed for a few seconds, centrifuged at 12,000 rpm [21]. The supernatant was mixed with chloroform and centrifuged. The DNA was precipitated with 3.0 M sodium acetate (Merck, Germany) and an equal volume of isopropanol (Merck, Germany) at − 20 °C for 10 min, washed with 70% ethanol, then dried and suspended in 50 μl of double-distilled water. The final solution was kept at − 20 °C until used as a template for PCR [19]. For molecular identification of common dermatophytes, the PCR mixture was produced containing Taq DNA Polymerase 2 × Master Mix RED with 2 mM MgCl_2_ (Amplicon, Denmark), and 30 pmol of each ITS1 (5′TCCGTA GGTGAACCTGCG G-3′) and ITS4 (5′-TCCTCCGCT TATTGATATGC-3′) primers and extracted DNA (3 μl) in a final volume of 50 μl. The PCR conditions were according to Ghojoghi et al*.* [22]. The amplified products were visualized by electrophoresis after running in 1.5% agarose gels for one hour. To identify at a species level, the PCR products were subjected to digestion with the restriction enzyme *Mva*I. The total volume of PCR mixture was 32 μL contained 10 μL of PCR product, 2 μL of 10X buffer, 2 μL of the enzyme, and 18 μL nuclease free-water as the manufacturer (Thermo Scientific, USA). Reactions were incubated at 37 °C in a dry oven for 16 h and electrophoresed in a 2% agarose (CinnaGen, Iran) gel. The gels were analyzed under UV light. Final Molecular identification was performed by sequencing and BLAST (http://www.ncbi.nlm.nih.gov/BLAST/) from the National Center for Biotechnology Information (NCBI) database. Each species was identified from the best-scoring reference sequence of the blast output with an identity > 98% compared to the query sequence.

### Antifungal susceptibility studies

The antifungal susceptibility test for each isolate to terbinafine (TER), griseofulvin, caspofungin (CAS), fluconazole (FLU), itraconazole (ITR), luliconazole, and isavuconazole was performed, according to the microdilution CLSI M38-A2 and CLSI M61 standard methods [19, 20]. The powders of all antifungal agents were obtained from the manufacturers (Sigma, Germany). The concentration ranges of FLU, ITR, CAS, TER, and GRI in the wells was 0.016 to 8 μg/ml while the range for LUL and ISA was 0.008 to 4 μg/ml.

The isolated species were cultured on Potato Dextrose Agar (Himedia) and stored for 5 to 7 days at 35 °C. The conidia suspension was prepared in distilled water containing 0.025% tween 20 (Sigma-Aldrich). It was allowed to settle for 5 min to remove heavier particles. The conidial inoculum suspensions were adjusted spectrophotometrically (Pharmacia biotech Cambridge, England ultrospec 3000 UV/visible spectrophotometer) to optical densities ranging from 0.09 to 0.11 and diluted 1:50 in RPMI (Sigma, St. Louis, Missouri). Final suspension was made 2 × more concentrated than the density needed for testing (1 × 10^3 ^− 3 × 10^3^ CFU/mL) by plating 0.01 mL of the adjusted inoculum on Sabouraud dextrose agar to determine the viable number of colony-forming units (CFU) per milliliter. Antifungal agents diluted with RPMI‐1640 medium with pH 7.0 (corrected by morpholinopropanesulphonic acid, Sigma, USA) according to the manufacturer. Positive and negative controls were prepared by the wells without antifungal agents and wells without fungi species. *Candida albicans* ATCC 10231 and *Candida parapsilosis* ATCC 22019 were used as quality controls in the same procedures. The MIC values of dermatophyte species to azole antifungal agents, GRI, and TER were considered as lower drug concentration with 80% or more reduction in growth compared to the growth in the drug-free medium. For CAS, the MEC value was evaluated microscopically and was the lowest concentration well that leads to the growth of small, compact hyphal, or rounded forms as compared to the hyphal growth seen in the drug-free control well.

### Statistical analysis

Data were collected in IBM SPSS Statistics for Windows, version 16. MIC_50_, MIC_90_ (concentrations that inhibited 50% and 90% of the isolates), and MIC geometric mean (GM MIC) values for each antifungal were calculated. Correlations between MIC values of antifungal agents were evaluated by the Pearson correlation test and were significant at a 0.05 level. The phylogenetic analysis was accomplished using the MEGA 7.0 software with the neighbor-joining method and Bootstrap analysis with 1000 replicates. The resulting tree was visualized and annotated using iTOLv6 [23].

## Results

Of the 3012 clinical samples of patients with suspected dermatophytosis during the study period, 207 fungi species similar to dermatophytes were isolated. By molecular assay, 198 isolates were positive for dermatophytes and 9 were non-dermatophytes species. Non-dermatophyte species were improperly diagnosed by routine method; however, they were re-identified by DNA sequencing with GenBank accession number as *Uncinocarpus reesii* (OM219066), *Penicillium chrysogenum* (OM219079), *Fusarium solani* (OM219626), *Pseudogymnoascus pannorum* (OM219627), *Engyodontium album* (OM219630), *Acremonium distortum* (OM108592), *Alternaria* species (OM756727), *Acremonium fusidioides* (OM756725), and *Fusarium species* (OM756730). Dermatophytes were collected from five university reference lab from Sari (50 isolates), Mashhad (47 isolates), Isfehan (43 isolates), Tehran (31 isolates), and Shiraz (27 isolates). Regarding the type of samples, 86.9% of the dermatophyte isolates (172/198) were recovered from the skin including 60 *Trichophyton mentagrophytes*, 54 *Trichophyton interdigitale*, 33 *Trichophyton rubrum, nine Microsporum canis, seven Trichophyton tonsurans* and nine other species (three *Epidermophyton floccosum*, three *Trichophyton simii*, two *Trichophyton violaceum*, and one *Trichophyton benhamiae*); 5.6% (11/198) from nails including six *Trichophyton mentagrophytes*, two *Trichophyton interdigitale*, two *Trichophyton tonsurans,* and one *Trichophyton rubrum*; and 7.6% from the hair (15/198 isolates) including 10 *Trichophyton mentagrophytes*, three *Trichophyton tonsurans*, one *Trichophyton interdigitale*, and one *Microsporum canis*.

**Fig. 1:**
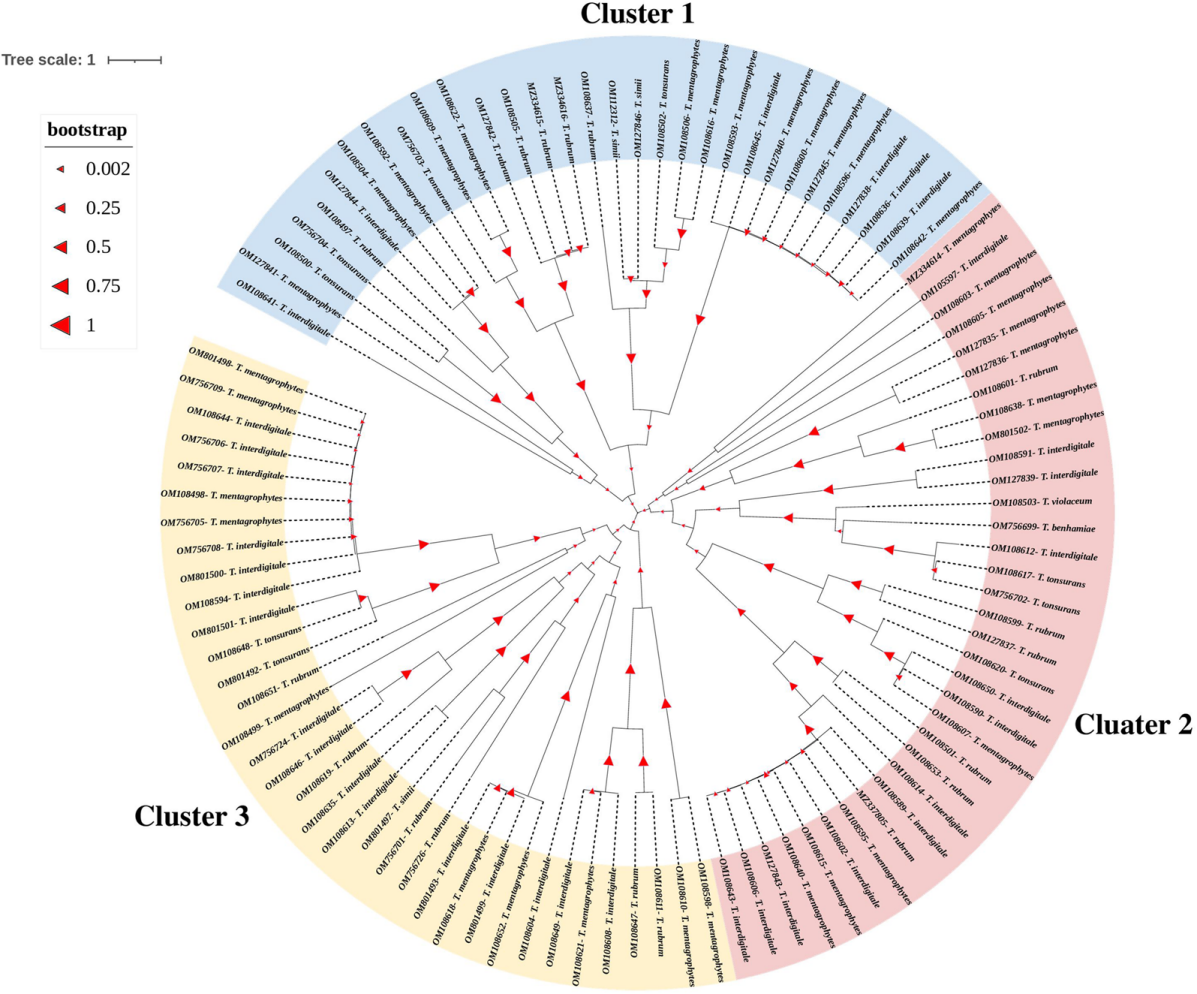
Phylogenetic tree of 100 representative *Trichophyton* species based on analysis of Internal Transcribed Spacer (ITS) sequences using the neighbor-joining method. ITS sequence accession numbers are given beside their name

The most commonly isolated dermatophyte species were *T. mentagrophytes* (77/198, 38.4%) followed by *T. interdigitale* (57/198, 28.8%), *T. rubrum* (34/198, 17.2%), and *T. tonsurans* (12/198, 6.1%). Other dermatophyte species identified in this study were: *Microsporum* (M) *canis* (10/198, 5%), *Epidermophyton floccosum* (3/198, 1.5%), *T. simii* (3/198, 1.5%), *T. violaceum* (2/198, 1%), and *T. benhamiae* (1/198, 0.5%). The amplified DNA of 100 dermatophyte isolates was subjected to sequencing and analyzed in the software Chromas (V. 2.6.2.). The phylogenetic analysis, accession numbers of the isolates, and the resulting tree was presented in Fig. 1. Given the low variability among ITS sequences of isolated dermatophytes, only three groups corresponding to the three genotypes were observed as closely related species. Both anthropophilic (*T. rubrum*, *T. tonsurans*, and *T. interdigitale*) and Zoophilic dermatophytes (*T. simii* and *T. benhamiae*) were found in a cluster.

**Fig. 2:**
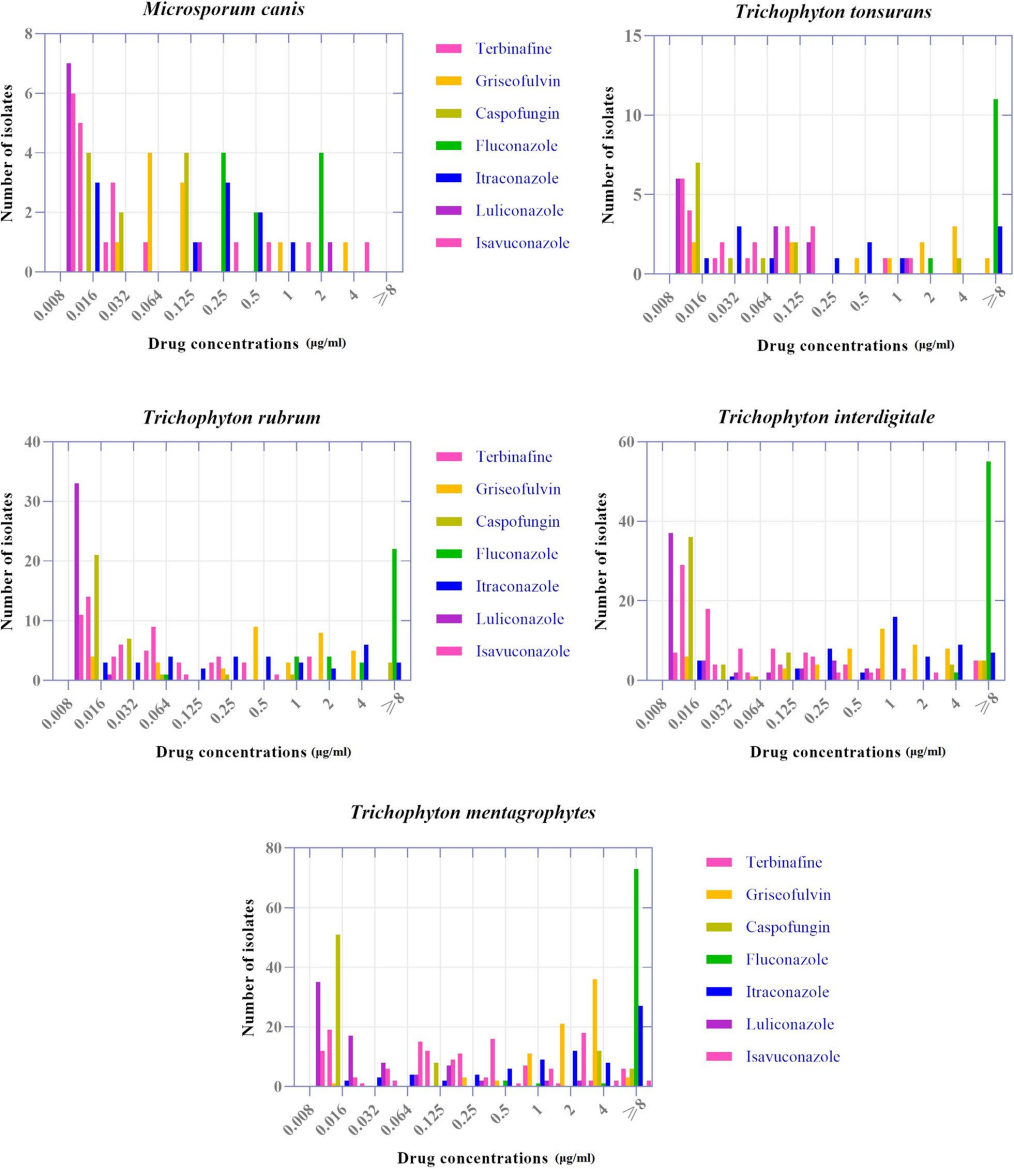
In vitro MIC90 values of dermatophyte isolates to seven antifungal agents by the CLSI broth micro-dilution test

The antifungal activity data of seven antifungal agents for dermatophyte species are presented in Tables 1, 2, and Fig. 2. There was not significant correlation between MIC values of antifungal agents in different cities. The GM MIC and MIC_90_ values for all the isolates were TER (0.091 and 1 μg/ml), GRI (1.01 and 4 μg/ml), CAS (0.061 and 4 μg/ml), FLU (16.52 and 32 μg/ml), ITR (0.861 and 8 μg/ml), ISA (0.074 and 2 μg/ml), and LUL (0.018 and 0.25 μg/ml). The GM MIC values of TER, GRI, CAS, FLU, ITR, LUL, and ISA for *M. canis* were 0.04, 0.171, 0.034, 1.36, 0.117, 0.02, and 0.025 μg/ml, respectively. The lowest MIC_90_ value of *T. mentagrophytes* was for LUL and ISA (0.125 μg/ml), followed by TER (2 μg/ml). *Trichophyton interdigitale* presented MIC_90_ values to TER, GRI, CAS, FLU, ITR, LUL, and ISA of 1, 4, 4, 32, 4, 0.25, and 0.5 μg/ml, respectively. The MIC_90_ values for LUL, TER, ISA, and CAS in *T. rubrum* were 0.008, 0.25, 0.5, and 1 μg/ml, respectively. *Trichophyton tonsurans* isolates presented the same MIC_90_ value for TER, CAS, LUL, and ISA (0.125 μg/ml). According to the data, among the tested antifungals, LUL and ISA presented lower and FLU and ITR presented higher MIC_90_ values against all dermatophyte species. Moreover, our results indicated LUL is significantly the most effective antifungal agent against dermatophyte isolates while GRI is not a reliable antifungal to treat dermatophytosis. Also, *T. mentagrophytes* and *T. interdigitale* (the most prevalent dermatophyte species) exhibited the highest resistance to the antifungal agents. There was a significant correlation between the MIC values of LUL with ITR, ISA, and GRF (p = 0.001), and also between ISA with CAS, ITR, LUL, and GRF (P < 0.05). There was no significant difference in drug susceptibility between the three clusters for the seven antifungal agents.

**Table 1:** Comparison of in-vitro activities of seven antifungal agents tested against *dermatophytes* species isolated from patients by CLSI method

Dermatophyte species No. (%)	Antifungal agents	Range (μg/mL)	MIC/MEC_50_^a^ (μg/mL)	MIC/MEC_90_ (μg/mL)	GM MIC^a^ (μg/mL)
*Trichophyton mentagrophytes*76 (38.4%)	Terbinafine Griseofulvin Caspofungin Fluconazole Itraconazole Luliconazole Isavuconazole	0.016–8 0.25–8 0.016–8 0.5–32 0.016–8 0.008–2 0.008–2	0.25 2 0.016 32 2 0.016 0.016	2 4 4 32 8 0.125 0.125	0.209 2.356 0.077 24.788 1.610 0.022 0.177
*Trichophyton interdigitale* 57 (28.8%)	Terbinafine Griseofulvin Caspofungin Fluconazole Itraconazole Luliconazole Isavuconazole	0.016–8 0.016–8 0.016–4 4–32 0.016–8 0.08–0.5 0.008–2	0.032 1 0.016 32 1 0.008 0.032	1 4 4 32 4 0.25 0.5	0.080 0.915 0.060 24.438 1.0142 0.019 0.049
*Trichophyton rubrum* 34 (17.2%)	Terbinafine Griseofulvin Caspofungin Fluconazole Itraconazole Luliconazole Isavuconazole	0.016–8 0.016–4 0.016–8 0.064–32 0.016–8 0.08–0.016 0.08–1	0.032 0.5 0.016 16 0.5 0.008 0.032	0.25 4 1 32 4 0.008 0.5	0.038 0.534 0.041 7.379 0.411 0.008 0.044
*Trichophyton tonsurans* 12 (6.1%)	Terbinafine Griseofulvin Caspofungin Fluconazole Itraconazole Luliconazole Isavuconazole	0.016–8 0.016–8 0.016–4 2–32 0.016–8 0.08–0.125 0.008–1	0.032 1 0.016 16 2 0.008 0.008	0.125 4 0.125 32 4 0.125 0.125	0.049 0.477 0.039 18.775 0.407 0.029 0.026
*Microsporum canis* 10 (5%)	Terbinafine Griseofulvin Caspofungin Fluconazole Itraconazole Luliconazole Isavuconazole	0.016–8 0.064–4 0.016–0.125 0.5–32 0.016–1 0.08–2 0.008–4	0.032 0.125 0.032 1 0.25 0.008 0.008	0.064 4 0.125 4 0.5 0.125 0.25	0.040 0.171 0.034 1.360 0.117 0.020 0.025
Species with less than 10 isolates^b^ 9 (4.5%)	Terbinafine Griseofulvin Caspofungin Fluconazole Itraconazole Luliconazole Isavuconazole	0.016–0.064 0.032–4 0.016–8 1–32 0.032–8 0.008–0.032 0.008–0.5	0.016 0.5 0.032 8 1 0.008 0.032	0.064 2 4 32 1 0.032 0.125	0.238 0.279 0.115 8.832 0.556 0.112 0.047
Total 198 (100%)	Terbinafine Griseofulvin Caspofungin Fluconazole Itraconazole Luliconazole Isavuconazole	0.016–8 0.016–8 0.016–8 0.064–32 0.016–8 0.08–2 0.008–8	0.064 2 0.016 32 1 0.008 0.064	1 4 4 32 8 0.25 2	0.091 1.010 0.061 16.524 0.861 0.018 0.074

^a^*MIC* minimum inhibitory concentration, *MEC* minimum effective concentration, *GM* geometric means

^b^included *Epidermophyton floccosum* (3 cases), *Trichophyton simii* (3 cases), *Trichophyton violaceum* (2 cases), and *Trichophyton benhamiae* (1 cases)

**Table 2:** The geometric means values and range for isolated species less than 10 by CLSI method

Dermatophyte species No. (%)	Antifungal agents	Range (μg/mL)	GM MIC (μg/mL)
*Epidermophyton floccosum* (3 cases)	Terbinafine	0.064–0.5	0.158
	Griseofulvin	2–4	4.00
	Caspofungin	0.016–4	0.1.00
	Fluconazole	32	4.00
	Itraconazole	0.5–4	1.58
	Luliconazole	0.008–0.125	0.02
	Isavuconazole	0.008–2	0.063
*Trichophyton simii* (3 cases)	Terbinafine	0.016	0.016
	Griseofulvin	0.064–2	0.357
	Caspofungin	1–8	0.358
	Fluconazole	32	32.00
	Itraconazole	2	1.00
	Luliconazole	0.008	0.008
	Isavuconazole	0.032–0.125	0.063
*Trichophyton violaceum* (2 cases)	Terbinafine	0.016	0.016
	Griseofulvin	0.032	0.032
	Caspofungin	2	0.016
	Fluconazole	1–2	1.41
	Itraconazole	0.032–0.064	0.045
	Luliconazole	0.008	0.008
	Isavuconazole	0.008	0.008
*Trichophyton benhamiae* (1 cases)	Terbinafine	0.016	0.016
	Griseofulvin	4	4.00
	Caspofungin	8	8.00
	Fluconazole	32	32.00
	Itraconazole	8	8.00
	Luliconazole	0.008	0.008
	Isavuconazole	0.5	0.50

The nucleotide sequences have been deposited in GenBank under the accession numbers MZ334614-16, MZ337805, OM105597, OM108497-506, OM108589-622, OM108635-53, OM112312, OM127835-46, OM756699-709, OM756724, OM756726, OM801492, OM801493, OM801497-502.

## Discussion

Dermatophytosis is one of the most common infectious diseases in humans, 20–25% of the fungal infections in the world are caused by dermatophytes [24]. This report presents the Iranian epidemiological data regarding the distribution of dermatophyte species isolated from five University labs over a period of 3 years by molecular methods. These techniques can help identify infecting species and monitor their distributions [22]. Also, sensitivity patterns of two new azole drugs were compared with the established drugs against dermatophytes. Utilizing sequencing methodology, the current study showed the most common dermatophyte isolates were *T. mentagrophytes*, *T. interdigitale*, and *T. rubrum*. In a recent study from Iran, the most frequent dermatophytes were *T. mentagrophytes* (20%), followed by *T. tonsurans* (10%), *T. rubrum* (6.7%), and *T. interdigital* (6.7%) [25]. In a study by Zamani and co-workers from Iran, *E. floccosum* (31%) was the most prevalent isolated species from specimens, followed by *T. rubrum* (26.2%) and *T. mentagrophytes* (20.3%) [26]. The most prevalent isolated species in Belgium was *T. rubrum* from all samplings [27]. In Eastern Saudi Arabia in a 20-Year survey, *Microsporum* species was the most common dermatophyte, accounting for 50.5% (n = 201) followed by *Trichophyton* species 36.9% (n = 147) [28]. The most common dermatophytes isolated from patients in Kuwait were *T. mentagrophytes* (39%), *M. canis* (16%), *T. rubrum* (10%), and *E. floccosum* (6.2%) [29]. In a systematic review of dermatophytes in Brazil, *T. rubrum*, *T. interdigitale*, and *T. mentagrophytes* were the most common species [30]. Morphological identification of dermatophyte species in cultures is uncertain due to colony variations and overlapping characters of the species. A broad differentiation in the distribution of dermatophytes exists between different geographic areas due to differences in lifestyle, socioeconomic and environmental conditions, humidity, and temperature [28, 31].

Dermatophyte species present various susceptibility to antifungal drugs [32]. There are reports regarding antifungal resistance in the treatment of tinea capitis, and TER-resistant *T. rubrum* in onychomycosis [33, 34], which can lead to unsuccessful or prolonged therapies with increased medical costs and possible side effects for respective patients. The MIC cutoff value of dermatophyte resistance to antifungal agents was not described but MIC ≥ 1 μg/ml was considered a cut-off value indicator in some reports [35, 36]. Drug resistance in dermatophytes has caused alternative ways such as the use of new antifungal drugs or the use of UV light (UV-A, UV-B, and UV-C) [37]. Luliconazole is an imidazole antifungal, also known as NND-502, synthesized by Nihon Nohyaku Co Ltd (Osaka, Japan), presenting strong in vitro antifungal activity against *Trichophyton, Candida*, *Aspergillus* species, melanized fungi and *Malassezia* species [38–41]. Isavuconazole is a novel broad-spectrum triazole agent with the same mechanism of action as the other triazoles. Koga et al*.* reported a very low MIC range against *Malassezia restricta* (0.004–0.016 μg/mL) [39]. Also, in Shokoohi et al. study, luliconazole demonstrated the lowest geometric mean MIC against black mold and melanized yeast [40]. The antifungal susceptibility profile of dermatophytes to these agents remains poorly examined. In the present study, the MIC90 values of LUL and ISA in all isolates were lower than other antifungal agents. GM MIC values of LUL against *T. interdigitale* and *T. mentagrophytes* isolates were 0.0016 and 0.0024 μg/mL, respectively, and GRF had the highest GM MIC value for *T. interdigitale* isolates (1.1 μg/mL) in Taghipour et al*.* [36]. In Wiederhold et al. (California, USA), the MIC_90_ value of LUL was lower than that of TER in *T. mentagrophytes* and *T. rubrum* isolates [41]. In Badali et al*.*, dermatophytes species collected from CBS Fungal Biodiversity Centre, Utrecht, the Netherlands, the MIC_90_s of all strains for ISA (2 μg/ml) were lower than that for ITR and CAS (4 μg/ml) and FLU (> 64 μg/ml) [42]. The MIC_90_ value of LUL on *T. rubrum* in China was reported to be 0.06 μg/ml [43]. In our study, LUL, ISA, CAS, and TER present more antifungal effects than other ones on *T. interdigitale* and in Behnam et al*.*, TER was the most effective antifungal agent [44]. Ghannoum and Isham reported that ISA had shown potent in vitro activity against dermatophytes and was more active than other triazoles tested (ITR and VOR) [45].

Previously, TER used to be a first-line antifungal treatment for *T. rubrum* in China and was reported the best antifungal agent for the treatment of dermatophytosis in Brazil, Iran, and the United States [41, 43, 44, 46, 47]. In the current study, TER was an active antifungal agent after LUL for all isolates. Also, the MIC_90_ value of TER (0.063 μg/ml) for all dermatophyte strains was the lowest in Badali et al*.* [42]. But, resistant species of *T. rubrum* with the MIC of > 4 μg/ml to TER were reported in Mukherjee et al*.* [34]. The MIC_90_ value of TER on *T. rubrum* in China and *M. canis* in Iran were reported 0.015 and 0.125 μg/ml, respectively [43, 44]. In 2015 in Mashhad, Iran, the MIC90 values of *T. mentagrophytes* to TER, GRI, and ITR were reported at 0.5, 4, and 2 μg/ml, respectively [44]. In Jiang et al*.* in China, the geometric mean MICs of *T. rubrum* isolates to TER, ISA, ITR, GRF, and FLU were 0.03, 0.13, 0.26, 1.65, and 2.12 μg/ml, respectively [43]. All *T. interdigitale* isolates in Taghipour et al*.* were susceptible to TER (MIC_90_ value = 0.0125 μg/mL) while the MIC values of TER for *T. mentagrophytes* isolates were in the range 0.007 −  ≥ 32 μg/mL [36].

Griseofulvin was the other first-line antifungal therapy for the treatment of dermatophytosis. In the present study, the MIC_90_ value of this agent in all isolates was high (4 μg/ml). It was less active than LUL, ISA, and TER against the isolated species. The MIC_90_ value of GRF in 111 T*. rubrum* clinical isolates was reported as 2 μg/ml [34]. Caspofungin, ITR, and FLU present lower activity than the two new triazoles (LUL, ISA) tested in the present study. Fluconazole presents the highest MIC_90_ value and lowest efficacy on all dermatophyte isolates. The MIC_90_ values of ITR and CAS in Badali et al*.* were reported as 4 μg/ml and for FLU was > 64 μg/ml [42]. The MIC_90_ value of FLU and ITR on *T. rubrum* were reported at 2 and 0.25 μg/ml, respectively in Behnam et al*.* [44]. These differences between the susceptibility patterns in the different studied isolates may be due to different geographical locations and environmental conditions which can affect the expression of related genes and production of specific enzymes in dermatophytes species.

In the present study, the whole genome analysis showed three clonal dermatophyte populations. It may explain the genetic homogeneity of the isolates. Drira and co-workers in Tanzanian reported that most of the 60 clinical isolates of *T. mentagrophytes* were complex and belonged to the anthropophilic variant [48]. Ungo-kore et al*.* in Northwest Nigeria reported in “phylogenetic analysis of dermatophytes fungi isolated from tinea capitis”, the phylogenetic tree of 28S rDNA sequences revealed a cluster consisting of anthropophilic and zoophilic [49]. Our study is similar to other studies [48, 49], indicating the isolates were complex and anthropomorphic and zoophilic species diverged from the same origin.

The limitation of this study was the limited number of isolates because we could not collect dermatophytes isolates from all university centers in Iran. If a minimum of 100 isolates from each isolate were entered in the study, we could calculate epidemiologic cut-off values for each fungus and antifungal drug. However, our study described and explored the MIC_90_ values of the prevalent dermatophytosis species to seven antifungal agents and the trend of their sensitivity to a variety of antifungal agents.

## Conclusion

According to our data, *T. mentagrophytes*, *T. interdigitale*, and *T. rubrum* were the most common fungal species isolated from the patients. Knowledge of these anthropophilic fungi can help develop strategies for the prevention and therapy of patients. LUL, TER, and ISA in vitro were shown to be the most effective antifungal agents against all dermatophyte isolates investigated. Identifying the etiologic agents of dermatophyte infections and evaluating their susceptibility patterns can help the efficient management of infection in high-risk patients.

## Data Availability

The datasets used and/or analyzed during the current study are available from the corresponding author on reasonable request.

## Abbreviations

GRI: Griseofulvin
ISA: Isavuconazole
LUL: Luliconazole
TER: Terbinafine
CAS: Caspofungin
FLU: Fluconazole
ITR: Itraconazole
MIC: Minimal inhibitory concentrations
